# Galectin-1 Restricts Vascular Smooth Muscle Cell Motility Via Modulating Adhesion Force and Focal Adhesion Dynamics

**DOI:** 10.1038/s41598-018-29843-3

**Published:** 2018-07-31

**Authors:** Min-Shao Tsai, Ming-Tsai Chiang, Dong-Lin Tsai, Chih-Wen Yang, Hsien-San Hou, Yi-Ru Li, Po-Chiao Chang, Heng-Huei Lin, Huan-Yuan Chen, Ing-Shouh Hwang, Pei-Kuen Wei, Chiao-Po Hsu, Kuo-I Lin, Fu-Tong Liu, Lee-Young Chau

**Affiliations:** 1 Institute of Biomedical Sciences, Academia Sinica, Taipei, Taiwan; 2 Institute of Physics, Academia Sinica, Taipei, Taiwan; 3 Research Center for Applied Sciences, Academia Sinica, Taipei, Taiwan; 4 Division of Cardiovascular Surgery, Department of Surgery, Taipei Veterans General Hospital, Taipei, Taiwan; 5 Genomics Research Center, Academia Sinica, Taipei, Taiwan

## Abstract

Vascular smooth muscle cell (VSMC) migration play a key role in the development of intimal hyperplasia and atherosclerosis. Galectin-1 (Gal-1) is a redox-sensitive β-galactoside-binding lectin expressed in VSMCs with intracellular and extracellular localizations. Here we show that VSMCs deficient in Gal-1 (Gal-1-KO) exhibited greater motility than wild type (WT) cells. Likewise, Gal-1-KO-VSMC migration was inhibited by a redox-insensitive but activity-preserved Gal-1 (CSGal-1) in a glycan-dependent manner. Gal-1-KO-VSMCs adhered slower than WT cells on fibronectin. Cell spreading and focal adhesion (FA) formation examined by phalloidin and vinculin staining were less in Gal-1-KO-VSMCs. Concomitantly, FA kinase (FAK) phosphorylation was induced to a lower extent in Gal-1-KO cells. Analysis of FA dynamics by nocodazole washout assay demonstrated that FA disassembly, correlated with FAK de-phosphorylation, was faster in Gal-1-KO-VSMCs. Surface plasmon resonance assay demonstrated that CSGal-1 interacted with α5β1integrin and fibronectin in a glycan-dependent manner. Chemical crosslinking experiment and atomic force microscopy further revealed the involvement of extracellular Gal-1 in strengthening VSMC-fibronectin interaction. *In vivo* experiment showed that carotid ligation-induced neointimal hyperplasia was more severe in Gal-1-KO mice than WT counterparts. Collectively, these data disclose that Gal-1 restricts VSMC migration by modulating cell-matrix interaction and focal adhesion turnover, which limits neointimal formation post vascular injury.

## Introduction

Occlusive arterial disease caused by the formation of atherosclerotic plaque and intimal hyperplasia in the vessel wall is the leading cause of death worldwide^1^. It is well documented that upon vascular injury the vascular smooth muscle cells (VSMCs) undergo phenotypic transition resulting in increases in migration and proliferation and synthesis of extracellular matrix proteins to cause thickening of intima of vessel^2^. Compelling evidence has revealed the roles of growth factors, cytokines, extracellular matrix and mechanical forces in promoting the transition of VSMCs from the differentiated phenotype to proliferative phenotype during vascular injury and aging process^2^. The potential involvement of other factors in modulating the phenotypic remodeling of VSMCs remains to be explored.

Galectins are a family of β-galactoside-binding proteins with a wide spectrum of biological functions in a variety of cell types and tissues^3^. These proteins exhibit both extracellular and intracellular localizations^3,4^. It has been shown that extracellular galectins can modulate cellular functions through binding to surface receptors, extracellular matrix and adhesion molecules by their carbohydrate recognition domain^3^. Whereas intracellular galectins may influence cellular signaling and various biological processes through protein-protein interactions, which is independent of their glycan binding activity^4^. Galectin-1 (Gal-1) is a prototype galectin containing a single carbohydrate recognition domain and forms noncovalent dimer^5^. It contains six cysteine residues and is highly susceptible to oxidation^6,7^, resulting in the loss of lectin property but gaining new activity to promote macrophage activation and axonal regeneration^8,9^. Gal-1 is constitutively expressed in VSMCs and has been considered as a component of the vascular extracellular matrix^10,11^. It has been reported that Gal-1 expression is up-regulated in atherosclerotic vessels of humans and experimental animals^12,13^. Earlier studies have demonstrated that recombinant Gal-1 protein enhanced VSMC proliferation and modulated the attachment, spreading, and migration of VSMCs^11,12^. Nevertheless, the detailed mechanisms remained largely uncharacterized. Given that Gal-1 is a multifunctional protein, the potential roles of endogenous Gal-1 involved during VSMC remodeling post vascular injury deserve to be explored. To this end, in the present study we assessed the impacts of Gal-1 deficiency on platelet-derived growth factor (PDGF)-induced VSMC growth and migration responses using primary VSMCs isolated from wild type (WT) and Gal-1-knockout (KO) mice. Moreover, the effects of a redox-insensitive Gal-1 mutant protein, which was previously shown to preserve stability and glycan-binding activity^14^, as well as overexpression of WT-Gal-1 or a Gal-1 mutant defective in carbohydrate binding activity^15^ on VSMC proliferation and motility were examined. We further elucidated the underlying mechanism with particular interest focusing on Gal-1-mediated inhibition on cell motility. We also assessed the impact of Gal-1 deficiency on the development of intima hyperplasia in animal model.

## Materials and Methods

### Animals

The Gal-1-KO mouse line was originally obtained from Consortium for Functional Glycomics^16^ and backcrossed to C57BL/6 J genetic background. WT and Gal-1-KO mice were bred in house and kept on a 12 h light-dark cycle with free access to food and water. Sprague Dawley (SD) rats were provided by National Laboratory Animal Center, Taiwan. Carotid artery ligation was performed as described^17^. Briefly, WT and Gal-1-KO mice (male, 8–12 week of age) were anesthetized by intramuscular injection of a mixture containing ketamine (80 µg/g of body weight), xylazine (20 µg/g) and atropine (1.6 µg/g). The left common carotid artery was dissected after a midline incision of the neck and ligated with an 8-0 nylon suture at site just proximal of the carotid bifurcation. The incision was then closed with suture. At 4 weeks after ligation, both non-ligated and ligated carotid arteries were harvested, embedded in paraffin and sectioned (5 μm). The severity of intimal hyperplasia was assessed by Verhoff’s staining, and the intimal area and intima-media ratio at a distance of 400 μm from the ligation site were quantified. Animal experimental procedures were approved by the Institutional Animal Care and Utilization Committee of the Academia Sinica, Taiwan, and performed in accordance with the relevant guidelines and regulations.

### Primary VSMC isolation and culture

Mice (WT and Gal-1-KO) and SD rats of 8–10 week of age were anesthesia with 5% chloral hydrate and perfused with phosphate buffer saline (PBS) to clear blood from vessels. After the thoracic aorta was harvested and adventitia removed, the vessel was cut into small pieces, and digested with collagenase type II (300 U/ml; Sigma) in Dulbecco’s modified Eagle’s medium (DMEM; Gibco) containing 10% fetal bovine serum (FBS; Gibco) for 1 h at 37 °C with gentle rotation. After digestion, vascular cells were collected and washed twice with DMEM containing 10% FBS by centrifugation at 300 × g for 5 min, re-suspended in fresh DMEM containing 10% FBS and transferred to 6 cm dish. Culture medium was changed every 2 days. After reaching confluency, cells were trypsinized and reseeded on 10-cm dish for expansion. We confirmed that over 95% of cells isolated were α-SMA^+^-VSMCs (Supplementary Fig. 1). For comparative studies of WT and Gal-1-KO VSMCs, 3 mice of each genotype were used for each cell isolation to avoid variations among individual animals. Cells of the third passage were used for all *in vitro* experiments.

### Establishment of rat VSMCs with ectopic overexpression of Gal-1

The bicistronic IRES-yellow fluorescent protein (YFP) retroviral vectors bearing Flag-tagged human Gal-1 cDNA and Gal-1 mutant cDNA with a mutation at W69G (Gal-1-W69G), respectively, were prepared as described previously^15^. The primary rat VSMCs (2^nd^ passage; 2 × 10^5^) were seeded in 6-well plate for 24 h. Media were then replaced by 2 ml DMEM containing 10% FBS, retrovirus (MOI: 200) and 8 µg/ml polybrene, followed by centrifugation at 920 × g at 37 °C for 1 h. After incubation for 24 h, media were replaced by DMEM containing 10% FBS and cells were further incubated for 24 h in culture. Cells were then harvested and YFP-positive cells were sorted by FACSAria (BD Biosciences) and expanded in culture. Cells with passage number less than 8 were used for experiments.

### Recombinant CSGal-1 protein preparation

A cysteine-less mutant of human Gal-1 construct (CSGal-1)^14^ was subcloned into pET25b vector. The tag-free recombinant Gal-1 protein was induced in E. coli BL21 (DE3) cells cultured overnight with 1 mM isopropyl-1-thio-β-D-galactopyranoside. Bacteria pellets were collected by centrifugation, resuspended in buffer containing 20 mM Tris-HCl pH 7.5, 5 mM EDTA, 1 mM dithiothreitol, and protease inhibitor cocktail, and lysed by sonication at 4 °C as described previously^18^. CSGal-1 protein was then purified from lysates by lactosyl-Sepharose 4B affinity chromatography. After extensive wash with PBS, CSGal-1 protein was eluted with 250 mM lactose, dialyzed against PBS and sterilized by filtration prior to storage at −80 °C.

### Proliferation assay

Mouse and rat VSMCs (3 × 10^4^) were seeded in 12-well plate. Next day, mouse and rat VSMCs were subjected to serum deprivation in serum free DMEM for 24 h and 48 h, respectively. Cells were then treated with medium containing indicated concentrations of FBS or PDGF. Each treatment was performed in triplicate. At 48 h, cells were harvested and counted by trypan blue exclusion method.

### Migration assay

VSMC migration was assessed by wound healing and Boyden chamber migration assays. For wound healing assay, VSMCs (1 × 10^4^) were seeded in triplicate on both sides of a culture-insert (Ibidi, Munich, Germany) with a 500 μm gap between each side of the well, and grew for 24 h to reach confluency. Following serum deprivation as described above, the insert was removed and cells were incubated in DMEM with or without 10 ng/ml PDGF. Cells were photographed at insert removal (0 h) and after 8 or 24 h of incubation as indicated. The results were quantified by Image J software (NIH). For Boyden chamber migration assay, serum-starved VSMCs (3000 cells/chamber) suspended in DMEM containing 0.1% FBS were seeded in the upper chamber of multi-well chemotaxis chamber with 8 μm-pore filter membrane coated with 0.1% gelatin. The bottom chamber was filled with DMEM containing 10 ng/ml PDGF as chemoattractant. After 5 h incubation in culture, cells migrated through membranes were fixed in methanol for 15 min and stained 30 min with Giemsa’s solution. Cells were then photographed and counted.

### Matrix adhesion assay

The 96-well plate was pretreated with different substrates (10 μg/ml) in Hank’s balanced salt solution (HBSS) at 37 °C for 2 h. Cells were harvested, resuspended in serum free DMEM at a density of 2 × 10^5^/ml and transfer (100 μl) to triplicate wells. After incubation at 37 °C for 1 h, non-adherent cells were removed by 3 washes with HBSS, and attached cells were fixed with 4% paraformaldehyde, washed and stained with Crystal Violet (1 mg/ml) for 30 min at room temperature. The plate was washed 4 times with distilled water and dye was dissolved out in 150 ul of 20% acetic acid. After incubation at room temperature for 30 min, 100 ul was removed into a new plate, and the absorbance at 560 nm was determined. To measure real-time adhesion dynamics, a modified nanoslit surface plasmon resonance (SPR) sensor system was used^19^. Briefly, the biochip surface was coated with 10 μg/ml fibronectin (FC010, Chemicon). VSMCs (6.5 × 10^4^) in 130 ul of DMEM were applied to the SPR sensing biomicrofluidic chip. The chip was then placed on a computer controlled motor stage of microscope (IX71, Olympus). The temperature of the chip (37 °C) was sensed by a K-type thermocouple (TPK-02A, TECPEL) clipped between the chip and the indium tin oxide glass (ITO glass, Part No. 300739, Merck) heater and controlled by a proportional-integral-derivative (PID) controller (TTM-J40-R-AB, JETEC Electronics Co.). The optical wavelength change during the cell adhesion assay was recorded by the spectrometer (V2000, Ocean Optics) every 2 min and the total experiment period was 2 h. The cell adhesion kinetic curve was presented as the intensity changes of the specific spectra shift. The SPR resonant change was analyzed by spectral integration method as indicated in the equation with a slight modification:

$$dA={\sum }_{\lambda ={\rm{500}}}^{{\rm{\lambda }}={\rm{600}}}|\frac{{\rm{{\rm I}}}(\lambda )-{{\rm{{\rm I}}}}_{O}(\lambda )}{{{\rm{{\rm I}}}}_{S}(\lambda )}|$$

Where dA is the SPR response, *I*_0_ (λ) is the intensity of the referenced spectrum (time = 0), *I*(λ) is the intensity of spectrum at indicated time point and *I*_s_ (λ) is a constant wavelength (substrate mode) served as the intensity internal control. The spectral integration region was selected from 600 nm to 650 nm, where the SPR occurred at the aluminum/medium interface. The data from 4 independent experiments were plotted and the curves were fitted (One phase association) with software Prism 6 (Graphpad). The value of rate constant was analyzed by software to compare the adhesion rate between WT and Gal-1-KO cells. The averaged data of cell adhesion percentage calculated by normalizing the time-indicated dA with the plateau dA.

### Cell spreading and focal adhesion analysis

Cells were plated on fibronectin (10 µg/ml)-coated glass coverslips for indicated times. Attached cells were fixed with 4% paraformaldehyde, followed by permeabilization with 0.2% Triton X-100 at room temperature for 10 min. After blocking with 2% bovine serum albumin (BSA) in PBS for 1 h, cells were stained with rhodamine-conjugated phalloidin (Molecular Probes) in dark for 30 min or incubated with mouse anti-vinculin antibody (sc-73614; Santa Cruz) at 4 °C overnight. Cells were then incubated with fluorescein isothiocyanate-conjugated anti-mouse antibody (GTX26785; GeneTex) at room temperature for 2 h. After 3 washes with PBS, stained cells were examined by epifluorescence microscope or confocal microscope as indicated. Cell size and focal adhesion number were quantified from images and analyzed with MetaMorph (Molecular Devices Corporation, Sunnyvale, CA).

### Nocodazole washout

Serum-starved cells were treated with 10 μM nocodazole in culture for 3 h. The drug was then washed out 4 times with serum free DMEM, and cells were incubated in serum free medium for various times in culture prior to fixation for confocal immunofluorescence staining or the preparation of cell lysates for Western blot analysis.

### Gal-1 binding assay

CSGal-1 was biotinylated by incubating 2 mg/ml of CSGal-1 in PBS with 2 mM EZ-link sulfo-NHS-LC-biotin (Thermo Scientific) at 4 °C for 2 h with rotation. Free biotin was removed by dialysis. To perform Gal-1 binding, VSMCs were washed twice with HBSS and then incubated with biotin-Gal-1(10 ug/ml) in the absence or presence of 100 mM lactose at 4 °C for 1 h. Cells were washed twice with HBSS and lysed with buffer containing 25 mM Tris-HCl pH7.5, 50 mM NaF, 1 mM Na_4_PO_7_, 1 mM NaV_3_O_4_, 0.5% NP-40, and protease inhibitor cocktail. After centrifugation at 12,000 × g for 10 min at 4 °C, the supernatant was harvested and protein concentration determined. The cell lysates (~700 μg proteins) were then incubated with 50 µl of streptavidin-conjugated magnetic beads (Cat.no. 65305; Invitrogen) for 2 h at 4 °C with rotation. Magnetic beads were then collected and washed 3 times with the same buffer. The bound proteins were eluted with 2 × SDS-sample buffer, followed by SDS-PAGE and Western blot analysis as indicated.

### Western blot analysis

Western blot analysis was performed as described previously^20^. The antibodies used are: anti-FAK (3285S, Cell Signaling), anti-phospho-FAK (3283, Cell Signaling), ant-Flag (F1804, Sigma), anti-β1 integrin (ab179471, Abcam), anti-α5 integrin (553319, BD Pharmingen^TM^), anti-Gal-1 (AF1245, R&D Systems), anti-GAPDH (ab8245, Abcam) and anti-β actin (GTX109639, Gene Tex).

### Crosslinking/Extraction

The crosslinking/extraction assay was performed as described previously^21^ with modification. Briefly, cells (1 × 10^5^) were seeded on fibronectin (10 ug/ml)-coated 3.5 cm-dish in HBSS for 16 h. Cells were washed 3 times with PBS and incubated with 1 mM 3,3 dithiobis [sulfosuccinimidylpropionate] in PBS at 4 °C for 30 min. The unreacted crosslinker was then quenched by incubation with 50 mM Tris-HCl for additional 15 min at room temperature. After 3 PBS washes, cells were extracted with PBS containing 100 mM lactose, 0.1% SDS, 0.5% Triton X 100 and protease inhibitor cocktail. After 3 PBS washes, the crosslinked proteins were recovered from dish by incubation with 50 mM dithiothreitol in PBS at 37 °C for 30 min. Both un-crosslinked and crosslinked proteins were then subjected to Western blot analysis.

### Enzyme-linked immunosorbent assay (ELISA)

The 96-well polystyrene high bind microplate (#9018; Corning) was incubated with 100 µl of 10 μg/mL recombinant mouse α5β1intergrin protein (7728-A5, R&D systems) in 50 mM Na_2_CO_3_ pH 9.6 at 4 °C overnight. After 3 washes with PBS, the remaining protein-binding sites were blocked with 5% BSA in PBS at room temperature for 1 h, followed by PBS wash. Various concentrations of biotin-CSGal-1 in HBSS together with or without 10 mM lactose or sucrose were then added to triplicate wells, followed by incubation at room temperature for 1 h. After 3 washes with HBSS, 100 µl of streptavidin conjugated to horseradish peroxidase (1:10000 dilution; N100, Thermo Scientific) in HBSS was added to each well and incubated for 20 min at room temperature. After 3 HBSS washes, horseradish peroxidase substrate was added into each well and color developed was determined by absorbance at 450 nm using a plate reader.

### Surface Plasmon Resonance (SPR) Assay

SPR measurement was performed at 25 °C using a Biacore T100 apparatus (Biacore GE Healthcare) in the Biophysics Core Facility, Department of Academic Affairs and Instrument service at Academia Sinica. An antibody capturing kit (GE Healthcare) was used to immobilize rat anti-mouse α5 integrin antibody (10 μg/ml; 553319, BD Pharmingen^TM^) onto CM5 chip (GE Healthcare) surface using N-hydroxysuccinimide and ethyl (dimethylaminopropyl) carbodiimide. The recombinant mouse α5β1intergrin (1 μg/ml) was then captured by the anti-α5 intergrin antibody. For experiment with fibronectin, fibronectin (10 μg/ml) was directly immobilized onto CM5 chip (GE Healthcare) surface using the same method as described above. Subsequently, serial concentrations of CSGal-1 were injected at flow rates of 30 μl/min to generate kinetics sensorgrams. To test the effect of lactose or sucrose, CSGal-1 was first pre-incubated with indicated concentration of lactose or sucrose at room temperature for 30 min prior to injection. The running buffer contained 10 mM HEPES pH 7.4, 150 mM NaCl, 3 mM EDTA, 0.0005% TWEEN-20. The analyte contact time was 60 sec and dissociation time was 120 sec. Regeneration contact time was 30 sec. The running buffer was used as the regeneration buffer for fibronectin-coated surface; whereas buffer containing 10 mM glycine, pH 2.5 was used as the regeneration buffer for α5β1intergrin-bound surface. Analysis of CSGal-1 binding to α5β1 integrin or fibronectin was performed by fitting to steady-state model using Biacore T100 evaluation software version 2.0.3. This model plots steady state binding response (R_eq_) against analyte concentration (C) to get equilibrium dissociation constant K_D_.

$$\begin{array}{c}{{\rm{R}}}_{{\rm{eq}}}={{\rm{CR}}}_{{\rm{\max }}}/{({\rm{K}}}_{{\rm{D}}}+{\rm{C}})+{\rm{RI}}\\ {{\rm{K}}}_{{\rm{D}}}={\rm{Equilibrium}}\,{\rm{dissociation}}\,{\rm{constant}}\,({\rm{M}})\\ {{\rm{R}}}_{{\rm{\max }}}={\rm{Analyte}}\,{\rm{binding}}\,{\rm{capacity}}\,{\rm{of}}\,{\rm{the}}\,{\rm{surface}}\,({\rm{RU}})\\ {\rm{RI}}={\rm{Bulk}}\,{\rm{refractive}}\,{\rm{index}}\,{\rm{contribution}}\,{\rm{in}}\,{\rm{the}}\,{\rm{sample}}\end{array}$$

### Atomic force microscopy (AFM)

The interaction force between VSMCs and fibronectin was determined according to the protocol previously reported^22,23^. A NanoWizard-3 AFM system (JPK Instrument AG, Berlin, Germany) mounted on an Zeiss inverted microscope (Carl Zeiss AG) was used for the measurements. The colloidal probe, which is 5 μm in diameter with nominal spring constant of 0.32~0.08 N/m (sQube, CP-PNPL-BSG-A-5), was first decorated with 10 mM polyethylene glycol (Sigma) for 5 min, followed by incubation with 0.25 mg/ml fibronectin for another 5 min. After PBS wash, the probe was immediately transferred into the AFM system. Before the measurement, cantilever was calibrated for the deflection inverse optical lever sensitivity and the spring constant by indentation in glass and thermal method, respectively, using the JPK Instrument software^24,25^. Serum-starved WT and Gal-1-KO VSMCs were randomly selected and indented at a site located at midway between the nucleus and cell margin and 10–15 force-distance curves in pixel array at different positions were collected. The probe repeatedly approached and retracted from cell surface at 900 nm/s piezo scanning speed and 3000 nm ramp size. AFM measurements were conducted at room temperature. To test the effect of CSGal-1, Gal-1-KO VSMCs were preincubated with 10 ug/ml of CSGal-1 in the absence or presence of 100 mM lactose in culture for 30 min prior to AFM measurement. The interaction force F of the probe tip with cell surface were estimated from the Hooke’s law:

$${\rm{F}}={\rm{k}}\cdot {\rm{\Delta }}z$$

Where k is the cantilever elastic spring constant; Δz is the tip displacement corresponding the flexural bending of AFM cantilever produced by the tip-sample interaction.

### Immunohistochemistry

Arterial sections were pretreated with retrieval solution (DakoCytomation) at 95 °C for 30 min, followed by incubation with 3% H_2_O_2_ for 15 minutes at room temperature to exhaust endogenous peroxidase activity. After blocking with 5% normal donkey serum (017-000-121, Jackson) plus 2% BSA in PBS containing 0.1% Tween-20 (PBST) for 1 h at room temperature, sections were incubated with normal goat IgG (sc-2028, Santa cruz) or goat anti-Gal-1 antibody (AF1245, R&D Systems) diluted in PBST at 4 °C overnight. After 3 washes with PBS, sections were incubated with anti-goat antibody conjugated with horseradish peroxidase (sc-2033, Santa cruz) at room temperature for additional 1 h, followed by PBS wash and antigen detection with 3, 3-diaminobenzidine. Sections were then counter-stained with hematoxylin.

### Statistical analysis

Quantitative data were expressed as mean ± SE of at least 3 independent experiments. Student’s t test was used for statistical analysis of data between two groups and one-way ANOVA was used for data more than two groups. *P* < 0.05 was considered statistical significance.

## Results

### Gal-1 deficiency enhances VSMC growth and migration ***in vitro***

**Figure 1:**
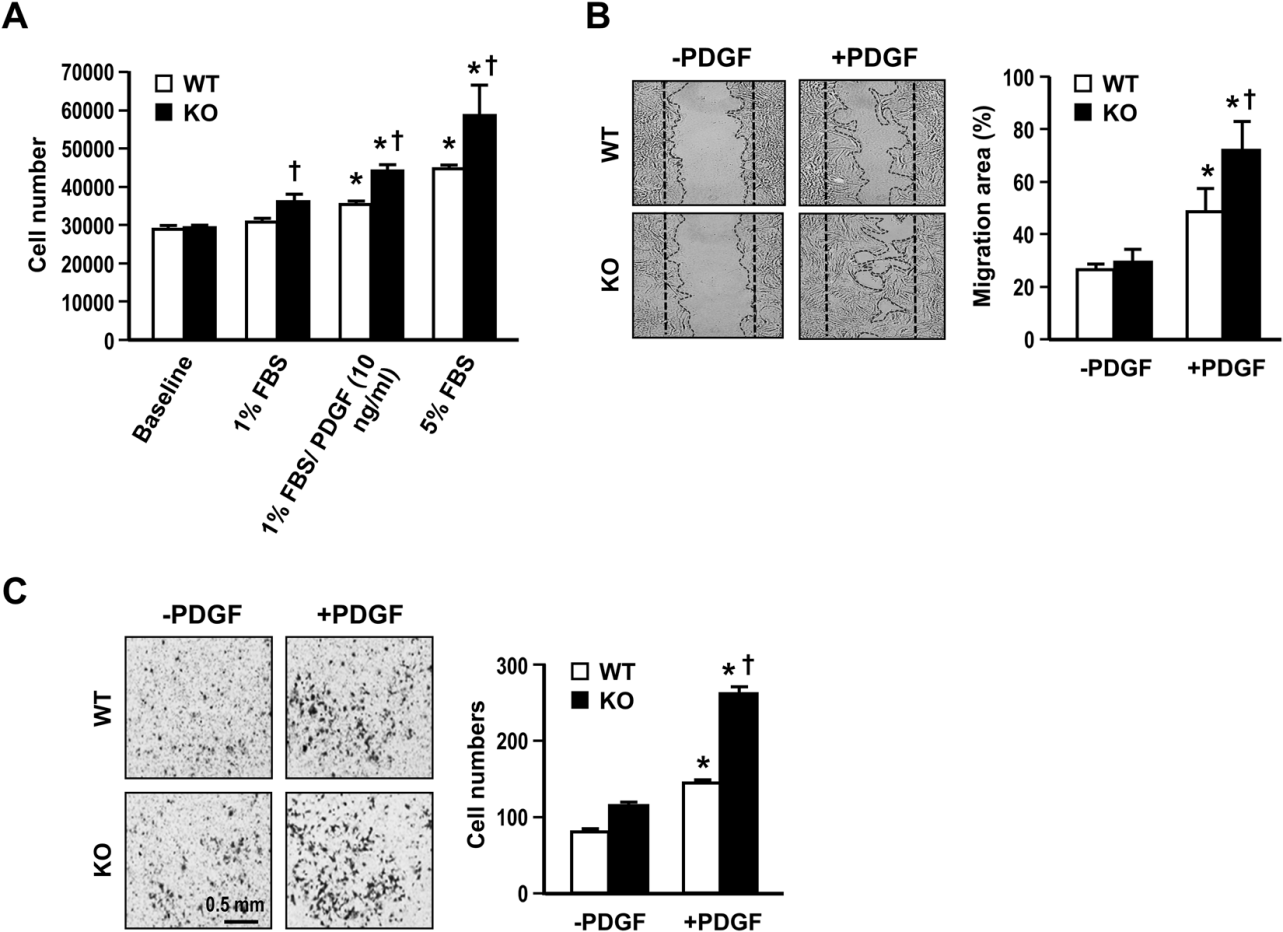
Gal-1 deficiency promotes VSMC proliferation and migration. (**A**) WT and Gal-KO VSMCs were subjected to serum deprivation for 24 h (baseline), followed by stimulation with indicated concentrations of serum or PDGF in culture for 48 h. The cell numbers were then determined. Data shown are mean ± SE of 3 independent experiments. *P < 0.05 vs 1%FBS; ^+^*P* < 0.05 vs WT VSMCs. Serum starved WT and Gal-KO VSMCs were subjected to PDGF (10 ng/ml) stimulation and the migration responses were assessed by wound healing assay (**B**) or Boyden chamber assay (**C**). (**B)** The representative photos showing VSMC migration to wound area at 8 h post stimulation with or without PDGF. The quantitative data are mean ± SE of 3 independent experiments. *P < 0.05 vs without PDGF; ^+^*P* < 0.05 vs WT VSMCs. (**C**) The representative photos showing migrated VSMCs at 5 h post stimulation with or without PDGF. The quantitative data are mean ± SE of 3-independent experiments. *P < 0.05 vs without PDGF; ^+^*P* < 0.05 vs WT VSMCs.

The phenotypic switch of VSMCs is a major event leading to intima lesion formation in injured vessel. To investigate whether Gal-1 has an impact on VSMC phenotype, we cultured primary VSMCs isolated from WT and Gal-KO mice and compared their growth rates and motilities *in vitro*. Both WT and Gal-1-KO VSMCs were serum deprived for 24 h (baseline) and then stimulated with 1% serum alone or together with PDGF (10 ng/ml) or 5% serum for 48 h. As demonstrated in Fig. 1A, the PDGF or serum-induced cell growth was 25~28% higher in Gal-1-KO VSMCs as comparing to that of WT cells. Experiment was also performed to examine the impact of Gal-1 on VSMC motility. As demonstrated in wound healing assay, PDGF-induced migration response was much greater in Gal-1-KO VSMCs comparing to WT counterparts (% migration area: 71.6 ± 10.7 vs 48.6 ± 8.8; *P* < 0.05) (Fig. 1B). Similar result was obtained when cell motility was assessed by Boyden chamber migration assay (migrated cell number: 261 ± 9 vs 144 ± 3; *P* < 0.05) (Fig. 1C).

### Gal-1 modulates VSMCs growth and migration depending on its lectin activity

**Figure 2:**
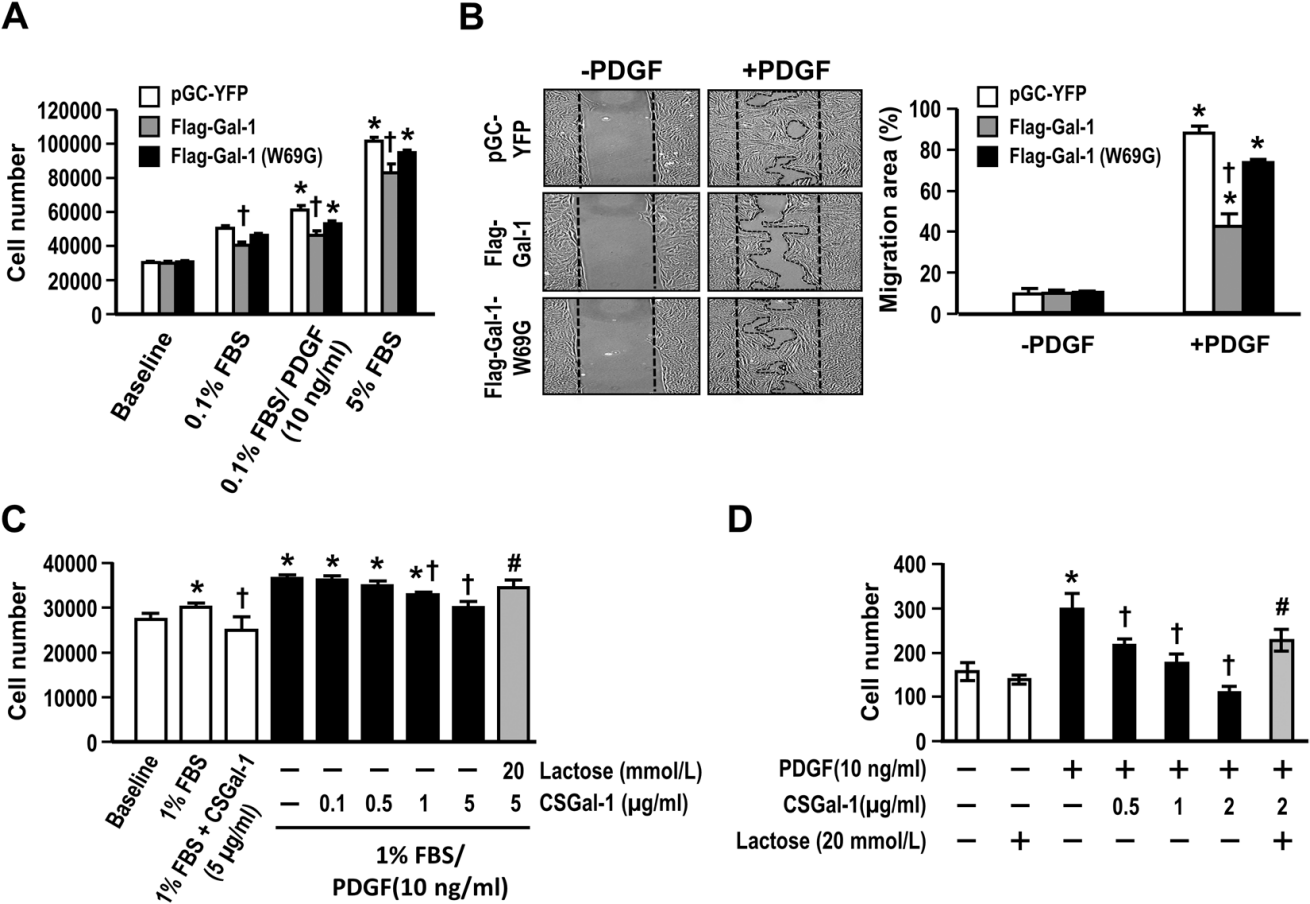
Gal-1 impacts VSMC proliferation and migration dependent on glycan-binding activity. (**A**) Serum-starved rat VSMCs without (pGC-YFP) or with WT Gal-1 or Gal-1-W69G overexpression as indicated were treated with PDGF or serum as indicated for 48 h. The increases in cell numbers were determined. Data are mean ± SE of 3 independent experiments. *P < 0.05 vs 0.1% FBS; ^+^*P* < 0.05 vs pGC-YFP control. (**B**) Serum starved rat VSMC lines as described above were subjected to wound healing assay. The representative photos were taken at 24 h after stimulation with or without PDGF. Quantitative data are mean ± SE of 3 independent experiments. *P < 0.02 vs without PDGF; ^+^*P* < 0.05 vs pGC-YFP control. (**C**) Serum-starved Gal-1-KO VSMCs were treated with 1% FBS together without or with PDGF (10 ng/ml) and indicated concentrations of CSGal-1 and lactose for 48 h. Cell numbers were then determined. Data shown are mean ± SE of 3 independent experiments. *P < 0.05 vs 1%FBS; ^+^*P* < 0.05 vs 1%FBS/ PDGF; ^#^*P* < 0.05 vs 1%FBS/PDGF/CSGal-1(5 µg/ml). **(D)** Serum-starved Gal-1-KO VSMCs were subjected to Boyden chamber migration assay with PDGF as chemoattractant. VSMCs were placed on upper chamber in the absence or presence of indicated concentrations of recombinant CSGal-1 and lactose. The quantitative data are mean ± SE of 3 independent experiments. *P < 0.05 vs without PDGF; ^+^*P* < 0.05 vs PDGF treatment alone; ^#^*P* < 0.05 vs PDGF/CSGal-1(2 µg/ml).

Gal-1 can modulate multiple cellular processes through both glycan binding-dependent and independent pathways. To evaluate whether the effect of Gal-1 on VSMC growth and migration is affected by its lectin activity, we established rat VSMC cell line with stable expression of WT Flag-tagged Gal-1 (Flag-Gal-1) or mutant protein bearing a glycine mutation at tryptophan 69 residue (Flag-Gal-1-W69G), which blocks its glycan binding activity^15^. To this end, the primary rat VSMCs were infected with a bicistronic IRES**-**yellow fluorescence protein (YFP) retroviral vector or vector bearing Flag-Gal-1 or Flag-Gal-1-W69G construct^15^. These infected cells coexpressed YFP protein and were sorted out by flow cytometer and expanded in culture. Western blot analysis demonstrated that the expression levels of Flag-Gal-1 and Flag-Gal-1-W69G in the respective cell lines were comparable (Supplementary Fig. 2). As shown in Fig. 2A, the growths of Flag-Gal-1-overexpressing cells induced by PDGF and serum were about 75.5% and 81.5% of control cells treated with the respective growth factors (*P* < 0.05). However, PDGF and serum-induced growth responses of Flag-Gal-1-W69G-overexpressing cells were 86.4% and 93.4%, respectively, of control cells, which did not reach statistical significance (Fig. 2A). The wound healing assay again showed that overexpression of Flag-Gal-1 resulted in substantial decrease in PDGF-induced cell migration (% migration area: 42.0 ± 6.2 vs 87.2 ± 3.3 (control); *P* < 0.05) (Fig. 2B). The migration was also reduced in VSMCs overexpressing Flag-Gal-1-W69G (73.2 ± 1.2% vs 87.2 ± 3.3 (control)), but to a much less extent. We further examined whether extracellular Gal-1 has effects on VSMC proliferation and migration. Given that Gal-1 is highly susceptible to oxidation to lose its glycan-binding activity^6,7^, we assessed the effect of a recombinant cysteine-less Gal-1 mutant protein (CSGal-1), which has been shown to preserve lectin activity and high stability^14^, on Gal-1-KO VSMCs. As shown in Fig. 2C, PDGF-enhanced growth was attenuated by CSGal-1 in a dose-dependent manner. Boyden chamber assay also showed that PDGF-induced migration response was markedly suppressed in the presence of CSGal-1 and completely blocked by CSGal-1 at 2 µg/ml (Fig. 2D). The inhibitory effects of recombinant CSGal-1 on PDGF-induced growth and migration could be reversed by coincubation with 20 mM lactose, a Gal-1 ligand, indicating that the effect of CSGal-1 is dependent on glycan-binding activity.

### Effect of Gal-1 deficiency on cell adhesion, spreading and focal adhesion assembly

**Figure 3:**
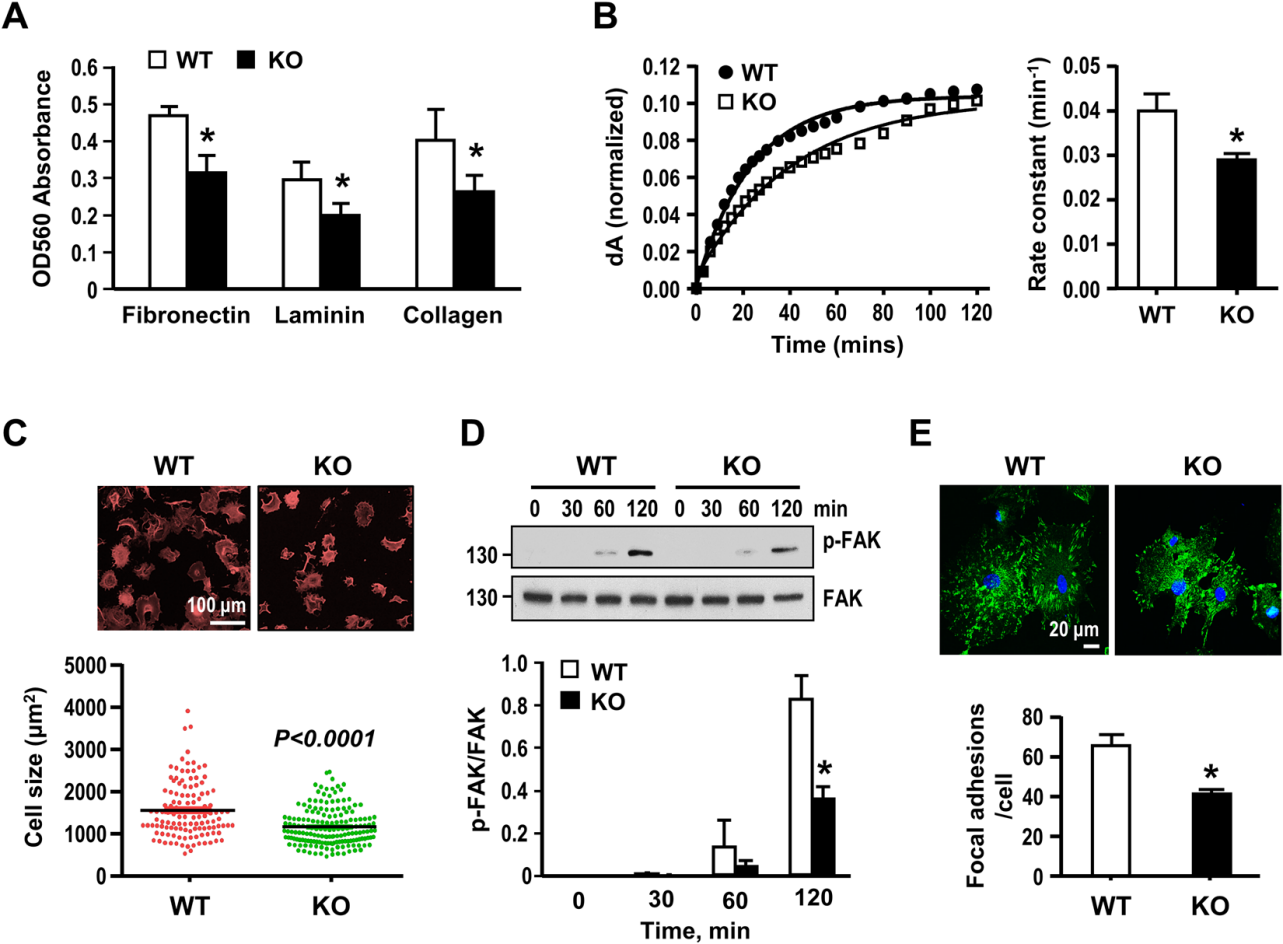
Gal-1 deficiency attenuates VSMC adhesion, spreading and focal adhesion formation. (**A**) Serum starved WT and Gal-1-KO VSMCs were plated on indicated matrix-coated 96-well plate for 1 h in culture. Nonattached cells were washed out and attached cells were stained with crystal violet and absorbance at 560 nm determined. Data are mean ± SE of 4 independent experiments. *P < 0.05 vs WT cells. **(B)** Serum-starved WT and Gal-1-KO VSMCs were applied to SPR sensing fibronectin-coated chip cell and the adhesion dynamics recorded for a period of 2 h. The time course plots showing the changes of SPR signal (dA) after normalization with the plateau dA in WT and Gal-1KO VSMCs. The values of rate constant shown are mean ± SE of 4 independent experiments. *P < 0.05 vs WT VSMCs. (**C**) Serum starved WT and Gal-1-KO VSMCs were plated on fibronectin-coated cover slips for 2 h. Cells were then fixed and stained with rhodamine-conjugated phalloidin. Sizes of spreading WT& Gal-1-KO VSMCs were quantified. (**D**) Serum-stared WT and Gal-1-KO VSMCs were plated on fibronectin-coated plates for indicated times. Cells were then harvested and FAK phosphorylation was examined by Western blot analysis. The quantitative data were mean ± SE of 3 independent experiments. *P < 0.05 vs WT cells. Uncropped images of immunoblots are shown in Supplementary Fig. 4A. (**E**) FA formation was examined by confocal immunofluorescence using antibody against vinculin. The FA numbers in WT and Gal-1-KO cells were quantified from 50~70 cells in each group compiled from 3 independent experiments. *P < 0.05 vs WT cells.

Given that cell-matrix interaction controls various cellular function and behavior, including proliferation and migration^26^, we then examined the adhesion abilities of WT- and Gal-1-KO VSMCs plated on matrix surfaces. As shown in Fig. 3A, Gal-1-KO-cells adhered less well than WT cells on fibronectin, laminin, and collagen. Assessment of adhesion dynamics by nano-slit surface plasmon resonance (SPR) sensor system^19^ again showed that Gal-1-KO-VSMCs adhered slower than WT VSMCs on fibronectin-coated surface (rate constant (min^−1^): 0.034 ± 0.003 vs 0.045 ± 0.002; *P* < 0.05) (Fig. 3B). When cell spreading was assessed at 2 h following plating on fibronectin, the result showed that Gal-1-KO-VSMCs displayed poorer spreading morphology with smaller cell size (Fig. 3C). Since focal adhesions (FAs) play a key role in regulating cell attachment and spreading^26^, the activation state of focal adhesion kinase (FAK), a key component of FAs^27^, following plating on fibronectin were examined. As revealed by Western blot analysis, phosphorylation of FAK (P-Y397) was significantly increased in adhered cells time-dependently (Fig. 3D). Moreover, the FAK phosphorylation reached a lower extent in Gal-1-KO-VSMCs. When the FA number was assessed by confocal immunofluorescence staining with a specific antibody against vinculin, a marker of mature FA, again Gal-1-KO cells have less FAs than WT cells examined at 2 h after plating on fibronectin (Fig. 3E). Together, these data disclose that Gal-1 facilitates cell-matrix interaction and initial FA assembly in VSMCs.

### Effect of Gal-1 deficiency on focal adhesion turnover

**Figure 4:**
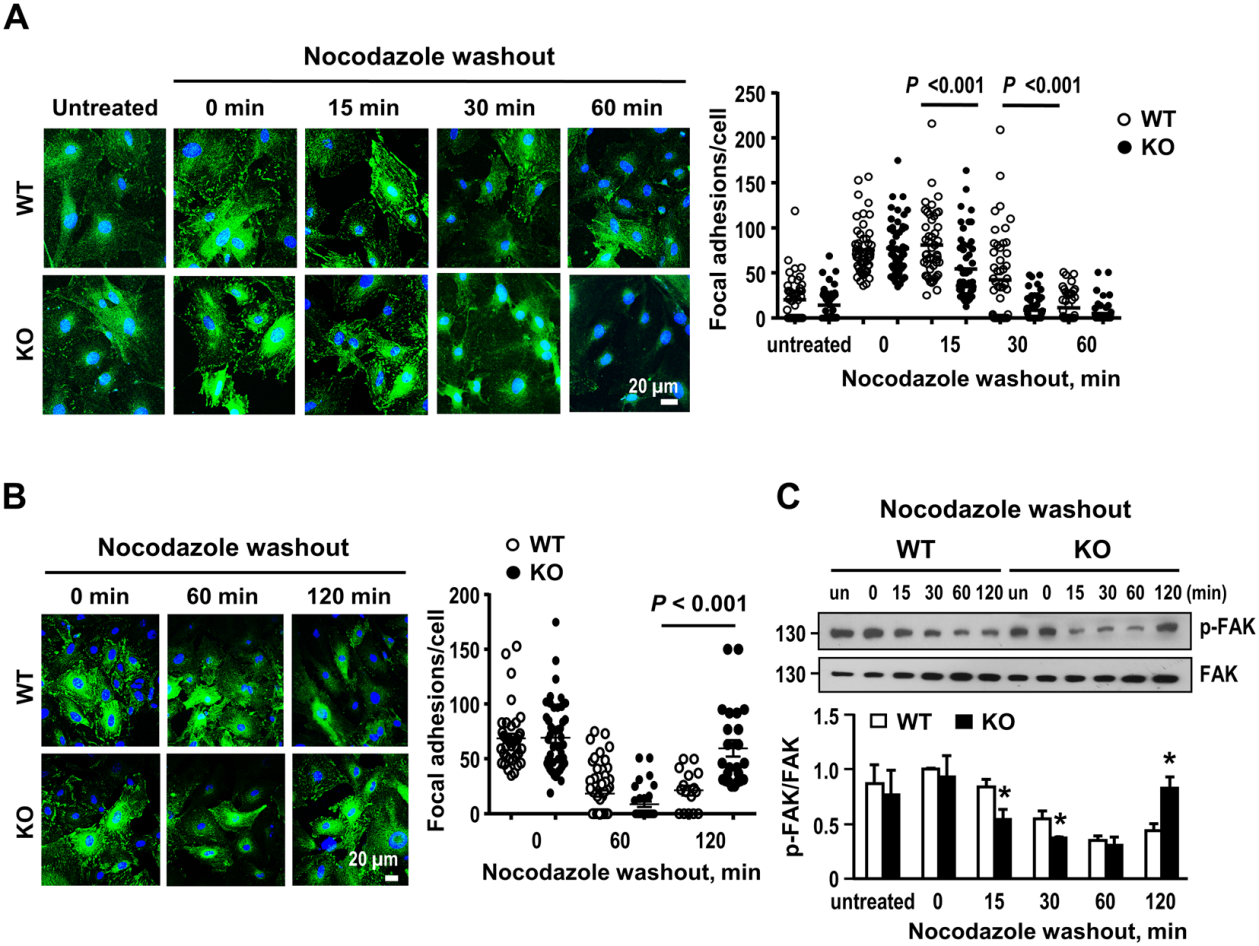
Gal-1 deficiency facilitates focal adhesion disassembly in VSMCs. (**A**) Serum starved WT and Gal-1-KO VSMCs were left untreated or treated with 10 μM nocodazole in culture for 3 h. Drug was then washed out extensively with serum free medium, and cells were incubated in serum free medium at 37 °C for indicated times. Cells were fixed and FAs were examined by confocal immunofluorescence staining using antibody against vinculin. The FA numbers from 10–20 cells/ genotype were quantified. Quantitative data show mean ± SE of 40–60 cells/genotype compiled from 3 independent experiments. (**B**) WT and Gal-1 KO VSMCs were subjected to nocodazole treatment as described above and the FA numbers at 1 and 2 h post drug washout were assessed. Data shown are the mean ± SE of 30–50 cells/genotype compiled from 2 independent experiments. (**C**) WT and Gal-1 KO VSMCs were subjected to nocodazole treatment for 3 h. Cells were harvested at indicated times post nocodazole washout and FAK phosphorylation was examined by Western blot analysis. Quantitative data are mean ± SE of 4 independent experiments.*P < 0.05 vs WT cells. Uncropped images of immunoblots are shown in Supplementary Fig. 4B.

Cell migration is controlled by FA assembly and disassembly^28^. To further investigate whether Gal-1 affects FA disassembly, we performed nocodazole washout experiment to assess the rate of FA disassembly in a synchronized manner^29^. Serum-starved cells were treated with nocodazole to destabilize microtubule polymerization. FA disassembly induced by microtubule regrowth was then assessed. As illustrated in Fig. 4A, the decrease in FA number was evident at 15 min and reached basal level at 30 min after nocodazole washout in Gal-1-KO cells. However, the FA disassembly in WT cells was slower and was not seen until 30 min after nocodazole washout. Likewise, the decrease of FAK phosphorylation after FA disassembly was faster in Gal-1-KO cells as shown in Fig. 4C. When FA was examined at 2 h post nocodazole washout, it was shown that FA reassembly was more prominent in Gal-1-KO cells (Fig. 4B). Again, the increase of FAK phosphorylation coincident with the FA reassembly at 2 h following nocodazole washout was also greater in Gal-1-KO cells (Fig. 4C). These results demonstrate that Gal-1 delays FA turnover in VSMCs.

### Analysis of Gal-1 binding to α5β1 integrin and fibronectin

**Figure 5:**
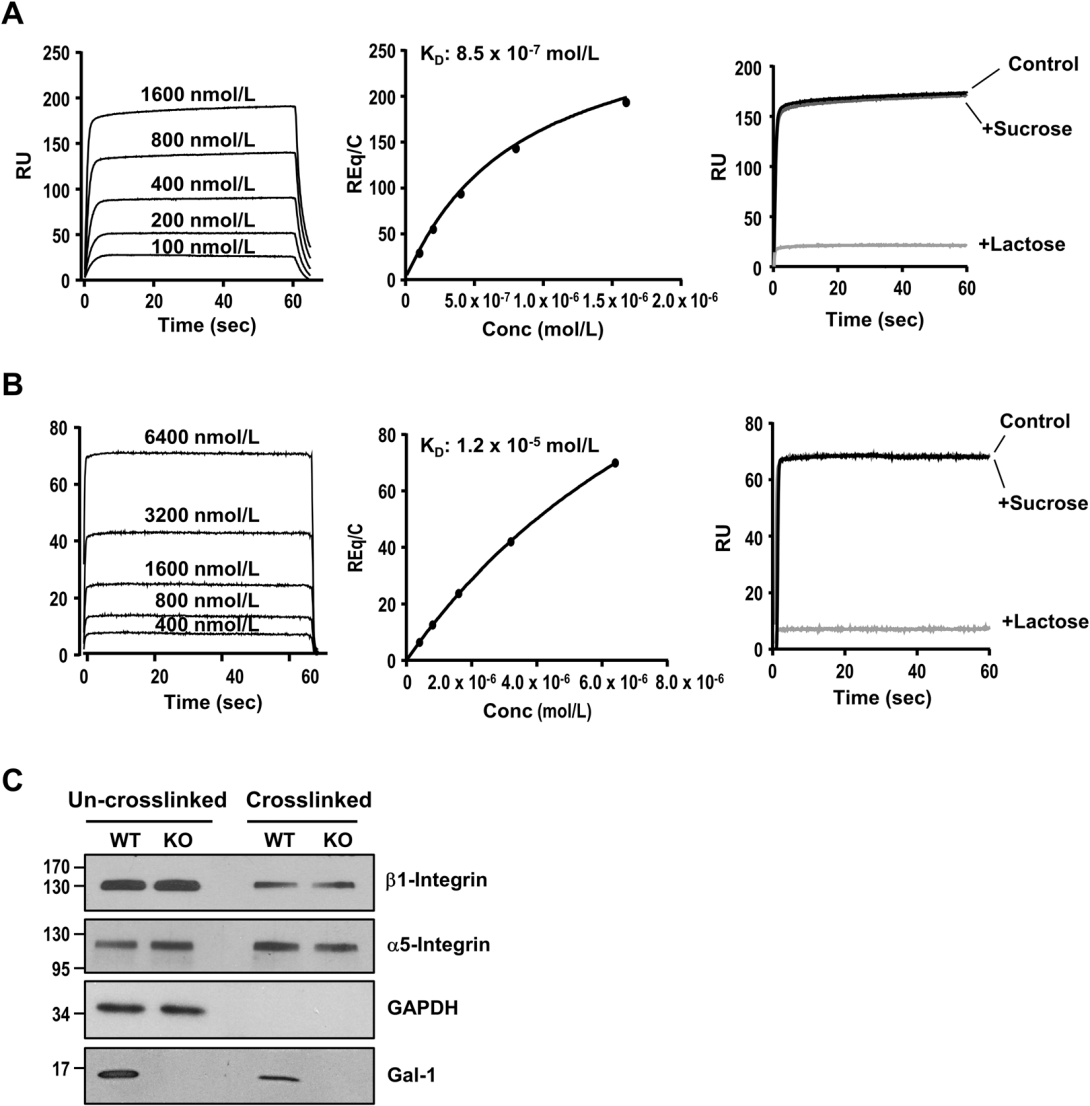
Characterization of interaction of Gal-1 with α5β1 integrin and fibronectin. **(A)** SPR analysis of interaction between CSGal-1 and α5β1intergrin. Recombinant mouseα 5β1intergrin protein was immobilized on a CM5 chip and CSGal-1 at indicated concentrations were injected at a flow rate of 30 μl/min. Left panel, sensorgrams generated with serial concentrations of CSGAL-1. Middle panel, the curve showing the specific signals obtained after subtracting the background binding to control chip without α5β1intergrin immobilization. Right panel, sensorgrams of CSGal-1 (1600 nM) pre-incubated without (control) or with 10 mM sucrose or lactose prior to injection. **(B)** Human plasma fibronectin was immobilized on a CM chip and CSGAL-1 at indicated concentrations were injected at a flow rate of 30 μl/min. Left panel, sensorgrams generated with serial concentrations of CSGAL-1. Middle panel, the curve showing the specific signals obtained after subtracting the background binding to control chip without fibronectin immobilization. Right panel, sensorgrams of CSGal-1 (6400 nM) pre-incubated without (control) or with 5 mM sucrose or lactose prior to injection. **(C)** WT and Gal-1-KO VSMCs were reseeded on fibronectin-coated dishes for 16 h. Cells were incubated with 1 mM 3,3-dithiobis[sulfosuccinimidylpropionate] in PBS at 4 °C for 30 min, followed by addition of 50 mM Tris-HCl to quench unreacted crosslinker. Cells were then extracted with PBS containing 100 mM lactose, 0.1% SDS and 0.5% Triton X-100. After 3 PBS washes, crosslinked proteins were recovered by incubation with 50 mM dithiothreitol in PBS at 37 °C for 30 min. Both un-crosslinked and crosslinked proteins were subjected to Western blot analysis with specific antibodies targeting respective proteins. Uncropped images of immunoblots are shown in Supplementary Fig. 4C.

Gal-1 has been shown to interact with β1 integrin in VSMCs^30^. To examine whether the interaction of extracellular Gal-1 with integrin contributes to its effect on FA turnover, we confirmed the binding of Gal-1 to β1 integrin by incubating WT-VSMCs with biotinylated CSGal-1. After cells were lysed and biotin-CSGal-1 pulled down by streptavidin-conjugated magnetic beads, Western blot analysis of the eluted proteins revealed that β1 integrin was co-eluted with biotin-CSGal-1, which was abolished by lactose cotreatment (Supplementary Fig. 3A). We then characterized the binding of Gal-1 with α5β1 integrin complex, the fibronectin receptor, by performing ELISA with recombinant mouse α5β1integrin-coated microplate. As shown in Supplementary Fig. 3B, biotin-CSGal-1 dose-dependently bound to recombinant α5β1 complex, which was abolished by co-incubation with lactose but not sucrose. We also performed SPR analysis to examine the binding kinetics between Gal-1 and α5β1 complex. As shown in Fig. 5A, CSGal-1 displayed a dose-dependent binding to α5β1 complex with fast association and dissociation rates. The calculated dissociation constant at the equilibrium (K_D_) was 8.52 × 10^−7^ M. Again the binding of CSGal-1 to α5β1 complex was abolished by lactose pretreatment, indicating it is glycan-dependent. We also assessed the interaction between CSGal-1 and fibronectin by SPR. The result showed that CSGal-1 bound to fibronectin with a K_D_ of 1.24 × 10^−5^ M, which was also dependent on glycan-binding activity (Fig. 5B). To further examine the binding of Gal-1 with fibronectin-integrin α5β1 complex in cellular context, we adopted the crosslinking/extraction method^21^ to detect proteins closely associated with fibronectin in cultured VSMCs. To this end, both WT and Gal-1-KO VSMCs were seeded on fibronectin-coated plate for 18 h in culture. After washing to remove non-adhered cells, the cell impermeable crosslinker, 3,3-dithiobis[sulfosuccinimidylpropionate] (12 Å spacer arm), was added to crosslink fibronectin with its integrin receptor and other interacting proteins. After extraction by buffer containing 100 mM lactose, 0.1% SDS & 0.5% Triton X-100 to remove the bulking cellular components, including intracellular Gal-1, and other unbound surface proteins, the bound proteins were recovered from the plate by dithiothreitol treatment and examined by Western blot analysis. As shown in Fig. 5C, both integrin β1 and α5, but not cytosolic glyceraldehyde 3-phosphate dehydrogenase, were detected in the de-crosslinked preparations, indicating that only proteins in close proximity with fibronectin could be crosslinked and retained on plate. Notably, Gal-1 was also detected in the de-crosslinked extract of WT VSMCs.

### Effect of Gal-1 on adhesion force between VSMCs and fibronectin

**Figure 6:**
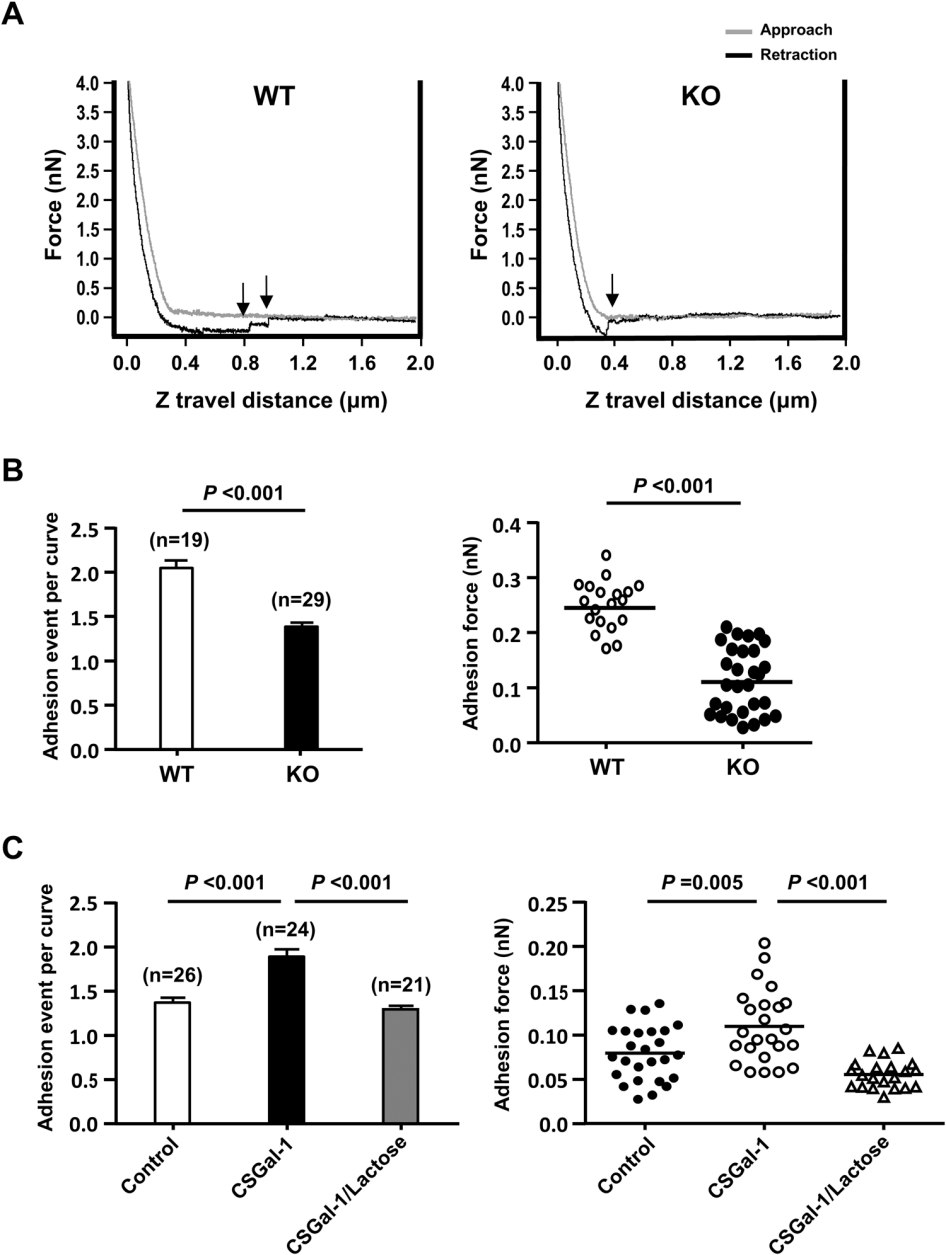
Gal-1 impacts the adhesion force between VSMCs and fibronectin. Fibronectin-coated AFM probe repeatedly approached and retracted from the surfaces of WT or Gal-1-KO VSMCs at 900 nm/s piezo ramp speed and 3000 nm ramp size. Data from 10–15 different contact sites/cell were collected and analyzed. **(A)** The representative force curves of VSMC-fibronectin interaction. Arrows indicate the events of adhesion rupture. (**B**) The numbers of rupture events during retraction and the adhesion forces detected in WT and Gal-1-KO VSMCs were determined. The numbers of cells for analysis in both genotypes are indicated in parentheses. (**C**) Gal-1-KO VSMCs were pretreated without (control) or with 10 µg/ml CSGal-1 in the absence or presence of 100 mM lactose in culture for 30 min prior to AFM analysis. The numbers of cells in various groups are indicated in parentheses.

To further assess whether Gal-1, which forms noncovalent homodimer, can enhance the binding strength of α5β1 integrin to fibronectin, we performed atomic force microscopy (AFM) to determine the adhesion force between fibronectin and surface of VSMCs as described previously by others^22,23^. The AFM cantilever with a round tip coated with fibronectin was used to repeatedly contact and retract from the surface of VSMCs at 10 randomly selected sites located at midway between the nucleus and cell margin. The adhesion force curves obtained clearly showed that less deflection shifts, which indicate the rupture of cell adhesions to fibronectin, were detected during the retraction from the surface of Gal-1-KO-VSMC surface than WT counterpart (Fig. 6A). The quantitative results showed that the adhesion events (the number of adhesion rupture) and adhesion force detected in Gal-1-KO-VSMCs were significantly less than those in WT VSMCs (Fig. 6B). To further confirm the role of Gal-1, Gal-1-KO VSMCs were preincubated with 10 ug/ml of CSGal-1 in culture for 30 min in the absence or presence of 100 mM lactose, followed by AFM analysis performed in the same medium. As shown in Fig. 6C, both adhesion events and adhesion force were significantly increased in CSGal-1-treated group. Moreover, the effect of CSGal-1 was abolished by lactose cotreatment as shown in the same figure.

### Intimal hyperplasia is augmented by Gal-1 deficiency in mice

**Figure 7:**
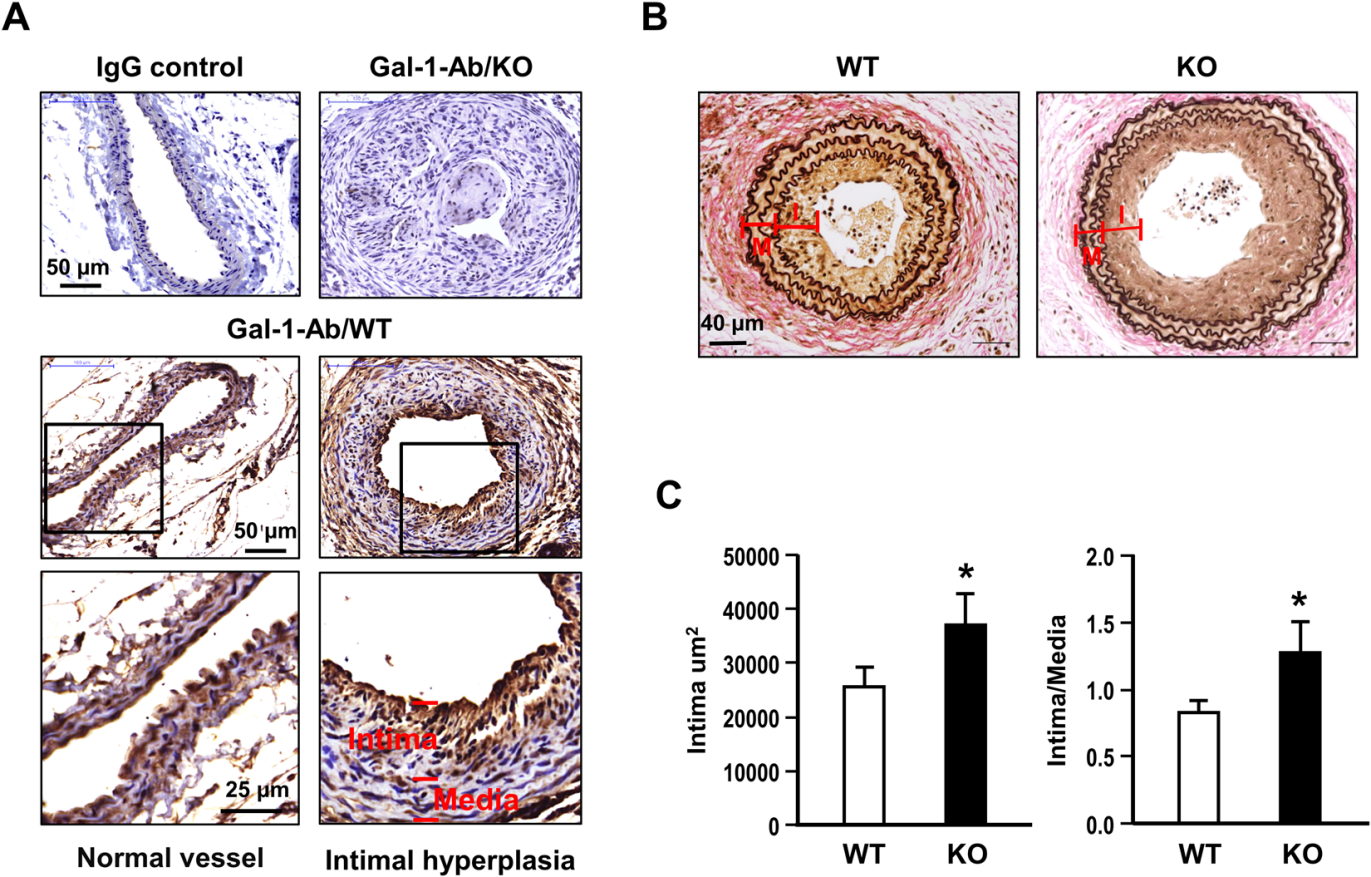
Gal-1 deficiency promotes neointimal formation in mice. Both WT (n = 11) and Gal-KO (n = 10) mice were subjected to carotid artery ligation for 4 weeks. (**A**) Gal-1 expression in arterial tissues of WT and gal-1-KO mice were examined by immunohistochemistry. (**B**) The representative Verhoff’s stains of vascular sections from WT and Gal-KO mice. (**C**) The quantitative results of the intimal area and intima-media ratio at a distance of 400 μm from the ligation site. *P < 0.05 vs WT group. I: intima; M: media.

To further explore whether Gal-1 deficiency impacts the vascular remodeling post vascular injury, we performed carotid ligation in Gal-1KO mice and WT controls. After 4 weeks post the ligation, the expression of Gal-1 and formation of neointimal lesions in these two groups of mice were examined. As demonstrated in Fig. 7A, Gal-1 expression as revealed by immunohistochemistry was evident in both endothelium and medial VSMCs of normal vessel in WT mice. Gal-1 expression was also prominent in the intimal VSMCs of injured vessel in WT mice but not detected in Gal-1-KO mice. When the formation of intimal lesion was evaluated by the increases of intimal area and intima-to- media ratio, both were increased to greater extents in Gal-KO mice as comparing to WT mice (Fig. 7B,C).

## Discussion

The present study demonstrated that mouse VSMCs deficient in Gal-1 exhibited greater proliferation and migration responses to PDGF. Overexpression of WT Gal-1 protein, but not the mutant Gal-1 defective in carbohydrate-binding activity, again led to significant reduction in PDGF-induced responses in rat VSMCS. Likewise, the redox-insensitive recombinant CSGal-1 suppressed PDGF-induced responses in Gal-1-KO VSMCs in a glycan-dependent manner. These observations support the importance of glycan-binding activity in Gal-1-mediated modulation of PDGF-induced VSMC phenotypic responses. Our findings, however, do not agree with previous reports by others showing that recombinant Gal-1 enhanced VSMCs proliferation and migration^11,12^. The discrepancies are likely caused by the differences in the experimental settings. Since the endogenous Gal-1 secreted to the extracellular compartment is susceptible to oxidative inactivation^6,7^, we used the activity-preserved CSGal-1 mutant protein rather than the WT Gal-1 protein for the *in vitro* experiments reported by others^11,12^. Furthermore, in contrast to the earlier studies in which the recombinant WT Gal-1-fusion protein was tested as an immobilized substrate coated on well/dish^11,12^, the soluble CSGal-1 protein was used in present studies. In any event, we observed that the effects of CSGal-1 are consistent with the results from the experiments comparing WT and Gal-1-KO VSMCs.

To elucidate the mechanistic details underlying Gal-1-mediated effects, we focused on the impact of Gal-1 deficiency on cell-matrix interaction, which controls various cellular function and behavior, including proliferation and migration^28^. Previous studies on various cell types have revealed the differential effects of Gal-1 on cell adhesion and migration^31,32^. Gal-1 has been shown to promote tumor progression through increasing cancer cell motility^33–37^. It also enhances dendritic cell^38,39^ and neutrophil^40^ migration, but inhibits T cell trans-endothelial migration^41^. Moreover, studies on endothelial cells demonstrated that Gal-1 facilitates angiogenesis via increasing cell proliferation and migration^42,43^. It is apparent that the effects of Gal-1 are cell-context specific. To understand how Gal-1 restricts cell motility in VSMCs, we first examined the adhesion abilities of WT and Gal-KO VSMCs on matrix proteins. Our experiments showed that Gal-1-KO-VSMCs adhered slower to matrix proteins with smaller spreading area and less FAs formed than WT cells, indicating that Gal-1 deficiency delays the cell-matrix interaction and FA assembly in VSMCs. Since cell motility is regulated by the dynamic turnover of FAs, the rates of FA disassembly in WT and Gal-KO VSMCs were also examined. As revealed by the nocadazole washout experiment, the FA disassembly in Gal-1-KO cells was much quicker than that in WT counterparts. These observations support the role of Gal-1 in modulating VSMCs motility through FA turnover.

The formation of FA is initiated by the binding of cell surface integrin receptors to extracellular matrix proteins. It has been shown that cell migration speed is affected by several variables, including the binding affinity of integrin-extracellular matrix^44^. In line with previous studies^11,12^, SPR assays demonstrated that CSGal-1 interacts with fibronectin as well as its receptor, α5β1 integrin, via glycan-dependent manner. Notably, the binding affinity of CSGal-1 to α5β1 integrin was much greater than that to fibronectin. This finding is consistent with previous study showing that Gal-1 binding to VSMCs was stronger than to extracellular matrix^12^. To further prove that endogenous Gal-1 secreted into extracellular compartment can interact with fibronectin and integrin complex in close proximity, we performed chemical crosslinking experiment with VSMCs plated on fibronectin-coated dishes using the cell impermeable crosslinker. The result clearly showed that both α5β1 integrin and Gal-1 were crosslinked with fibronectin. This observation raised the possibility that Gal-1 may influence the binding between integrin and matrix. Previous studies have shown that Gal-1 can either promote cell adhesion by cross-linking cell surface receptor and ECM or inhibit adhesion through competitive binding to cell surface receptor or matrix protein depending on different cell types and cellular contexts^31,45^. To explore whether the slower FA turnover and migration speed in WT VSMCs are resulted from Gal-1-mediated stronger binding force between integrin and matrix, we performed AFM to compare the adhesion force between WT and Gal-1-KO VSMCs. Our data clearly showed that the binding force between cells and fibronectin was significantly greater in WT VSMCs as compared to Gal-1-KO VSMCs. Pretreatment of Gal-1-KO VSMCs with CSGal-1 significantly enhanced the adhesion force. These results support the notion that Gal-1 restricts VSMC motility at least in part through enforcing the integrin-ECM interaction and slowing disassembly of FAs. However, given that Gal-1 is also present in the cytoplasm, the possibility that the intracellular Gal-1 has an impact on the stability of FA complex through modulating the signaling and binding of integrin to intracellular adaptor proteins and cytoskeleton cannot be completely ruled out in this study.

The impact of Gal-1 on vascular remodeling was demonstrated in the carotid artery ligation model. Gal-1 deficiency augmented the development of intimal hyperplasia in mice, supporting the role of ”Gal-1 involved in VSMC phenotypic modulation and remodeling process post vascular injury. The implication of Gal-1 in the regulation of VSMC function has also been reported previously by other groups. It has been shown that Gal-1 modulates vascular constriction via regulating the surface expression of Cav1.2 channel in VSMCS^46^. Moreover, Gal-1 deficiency influences vasocontractile response and remodeling in hypoxia-induced pulmonary hypertension in animals^47^. Taken together, these accumulating findings support the important functions of Gal-1 involved in the maintenance of vascular homeostasis.

## Electronic supplementary material

Supplementary Data
