# Supplementary material for: Galectin-1 Restricts Vascular Smooth Muscle Cell Motility Via Modulating Adhesion Force and Focal Adhesion Dynamics
Source: Sci Rep. 2018 Jul 31;8:11497. doi: 10.1038/s41598-018-29843-3 (PMC6068105; doi:10.1038/s41598-018-29843-3)
Supplement: Supplementary file 1 — Supplementary Data [file 41598_2018_29843_MOESM1_ESM.pdf]

Supplementary Information

Galectin-1 Restricts Vascular Smooth Muscle Cell Motility Via Modulating Adhesion Force and Focal Adhesion Dynamics

MS Tsai, MT Chiang, DL Tsai, CW Yang, HS Hou, YR Li, PC Chang, HH Lin, HY Chen, IS Hwang, PK Wei, CP Hsu, KI Lin, FT Liu, LY Chau

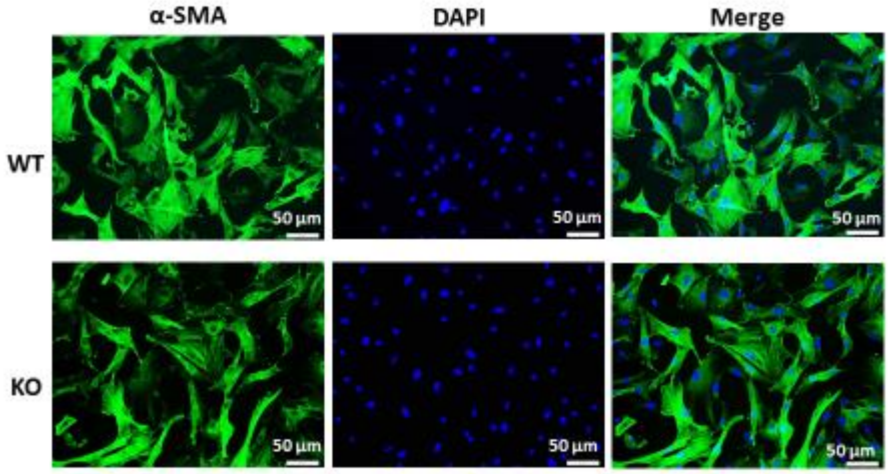

Supplementary Figure 1. The representative images of α-SMA immunofluorescence staining of VSMCs isolated from WT and Gal-KO mice. Primary VSMCs were isolated from mouse aortic tissues of WT and Gal-KO mice and cultured in DMEM medium containing 10% serum. The purities of cell populations were examined by performing immunofluorescence staining with specific antibody against α-SMA. The nuclei were counterstained with DAPI.

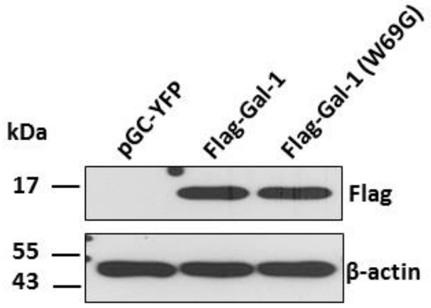

Supplementary Figure 2 Establishment of rat SCMC lines with stable overexpression of WT and mutant Gal-1. Primary rat VSMCs were infected with bicistronic IRES-YFP retroviral vector (pGC-YFP), vector bearing Flag-Gal-1 and Flag-Gal-1-(W69G) mutant constructs, respectively, for 48 h. Cells expressing YFP protein were sorted out and expanded in culture. The expression levels of Flag-Gal-1 and Flag-Gal-1-(W69G) in respective cells were examined by Western blot analysis.

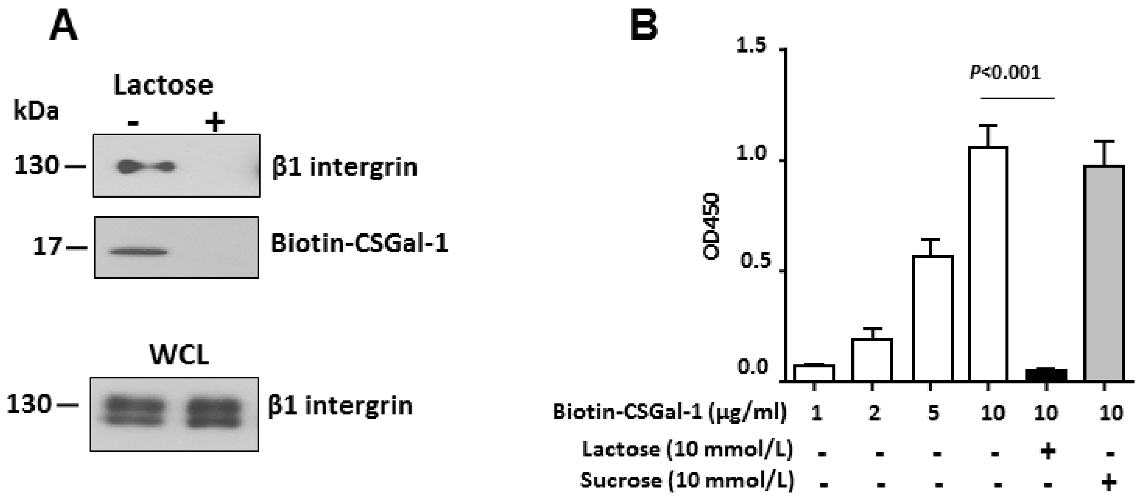

Supplementary Figure 3. Characterization of interaction between Gal-1 and α5β1integrin.
A Mouse WT VSMCs were incubated with biotin-CSGal-1(10 ug/ml) in the absence or presence of 100 mM lactose at 4°C for 1h. Cell lysates were prepared and biotin-CSGal-1 was pulled down by streptavidin-conjugated magnetic beads. Whole cell lysate (WCL) and eluted proteins were subjected to Western blot analysis with specific antibody against β1integrin. The eluted biotin-CSGal-1 was examined by incubation with streptavidin-conjugated horseradish peroxidase followed by detection with enhanced luminol-based chemiluminescent substrate. **B** Various concentrations of biotin CSGal-1 protein were incubated without or with lactose or sucrose as indicated in 96-well plate coated with recombinant mouse α5β1intergrin protein at room temperature for 1 h. After 3 washes, biotin-CSGal-1 bound to α5β1intergrin was detected by incubation with streptavidin-conjugated horseradish peroxidase, followed by peroxidase substrate reaction with absorbance at OD450 nm.

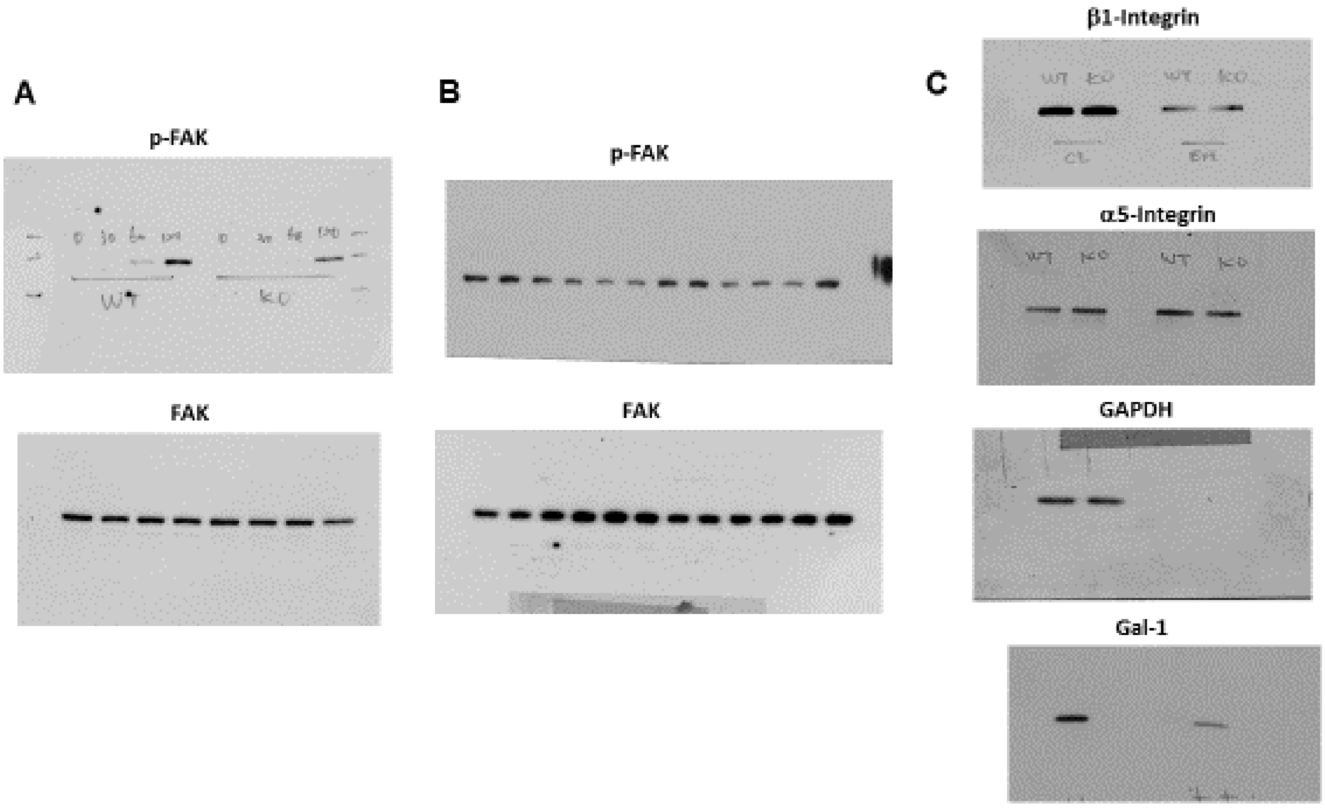

Supplementary Figure 4. Uncropped images of immunoblots shown in figures of main text.
